# Long non-coding RNA NR2F1-AS1: an increasingly significant LncRNA in human cancers

**DOI:** 10.1007/s13105-025-01119-1

**Published:** 2025-08-19

**Authors:** Qinfan Yao, Xinyi Zhang, Yitong Chen, Junhao Lv, Jianghua Chen, Dajin Chen

**Affiliations:** 1 Kidney Disease Center, the First Affiliated Hospital, College of Medicine, Zhejiang University, Qingchun Road 79, Hangzhou, 310003 China; 2 Key Laboratory of Kidney Disease Prevention and Control Technology, Zhejiang Province, Hangzhou, 310003 China; 3 National Key Clinical Department of Kidney Diseases, Hangzhou, 310003 China; 4 Institute of Nephropathy, Zhejiang University, Hangzhou, 310003 China; 5 Zhejiang Clinical Research Center of Kidney and Urinary System Disease, Hangzhou, 310003 China

**Keywords:** NR2F1-AS1, Cancer, LncRNA, Function, Clinical application

## Abstract

Long non-coding RNAs (lncRNAs), defined as transcripts exceeding 200 nucleotides without protein-coding potential, have emerged as pivotal regulators in diverse physiological and pathological processes, particularly in tumorigenesis. Among them, NR2F1-AS1, a recently characterized lncRNA, has garnered growing attention due to its dysregulated expression across a spectrum of malignancies and its significant correlation with key clinicopathological parameters. Accumulating evidence from molecular and cellular studies reveals that NR2F1-AS1 plays multifaceted roles in cancer initiation and progression through the modulation of signaling pathways, regulation of gene expression, and interactions with microRNAs and protein complexes. Notably, its biological function appears to be context-dependent: acting as an oncogene in many cancer types, such as breast, lung, liver, and gastric cancer, while exhibiting potential tumor-suppressive activity in others, including colorectal cancer, cervical squamous cell carcinoma, and thymic epithelial tumors. This review comprehensively summarizes the aberrant expression patterns, prognostic significance, biological functions, and molecular mechanisms of NR2F1-AS1, while also highlighting its emerging potential as a context-specific diagnostic biomarker and therapeutic target in human cancers.

## Introduction

Cancer encompasses a heterogeneous group of diseases characterized by uncontrolled cellular proliferation and the capacity for local invasion and distant metastasis [1–5]. Globally, cancer ranks as the second leading cause of mortality, following cardiovascular disease. According to the GLOBOCAN 2020 statistics, approximately 19.3 million new cancer cases were reported worldwide, with this number projected to escalate to 29.5 million by 2040 [5–8]. In light of the increasing global burden and generally unfavorable prognosis of malignant tumors, the discovery of effective and rapid-response biomarkers for early detection and the identification of novel therapeutic targets remain pressing clinical needs.

Long non-coding RNAs (lncRNAs), defined as transcripts longer than 200 nucleotides without protein-coding potential, have emerged as crucial regulatory molecules in gene expression [9–11]. Advancements in high-throughput sequencing technologies and bioinformatics tools have facilitated the identification of thousands of lncRNAs, many of which are now implicated in the pathogenesis of human diseases, especially cancer [12–16]. Of particular interest is the competing endogenous RNA (ceRNA) hypothesis, which proposes that lncRNAs can regulate mRNA expression by competitively binding to shared microRNAs (miRNAs), thereby exerting post-transcriptional control over critical signaling pathways [17–20]. In recent years, increasing evidence has highlighted the potential of lncRNAs as diagnostic, prognostic, and therapeutic biomarkers in oncology [21–24].

NR2F1-AS1 (nuclear receptor subfamily 2 group F member 1 antisense RNA 1), a recently identified lncRNA located on chromosome 5q15, spans approximately 171,965 base pairs. Aberrant expression of NR2F1-AS1 has been documented in a wide array of malignancies, including breast [25–27], gastric [28–31], bladder [32–34], thyroid [35–37], liver [38–41], osteosarcoma [42, 43], lung [44], esophageal [45, 46], pancreatic [45, 46], neuroblastoma [47], melanoma [48], endometrial [49], colorectal [50], cervical [51], and thyroid [52] cancers. Moreover, elevated levels of NR2F1-AS1 are significantly correlated with adverse outcomes, including poor overall survival, advanced TNM staging, larger tumor size, increased metastatic potential, recurrence risk, and resistance to therapy. Functionally, NR2F1-AS1 has been implicated in a variety of oncogenic processes such as cell proliferation, apoptosis evasion, migration, invasion, metabolic reprogramming (e.g., glycolysis), maintenance of cancer stemness, and chemoresistance.

Given its pervasive involvement across multiple cancer types and diverse regulatory functions, NR2F1-AS1 represents a promising candidate for biomarker development and targeted intervention. In this review, we provide a comprehensive overview of NR2F1-AS1, highlighting its expression patterns, clinical relevance, molecular mechanisms, and emerging roles in cancer diagnosis, prognosis, and therapy.

## The role of NR2F1-AS1 in human cancers

**Fig. 1:**
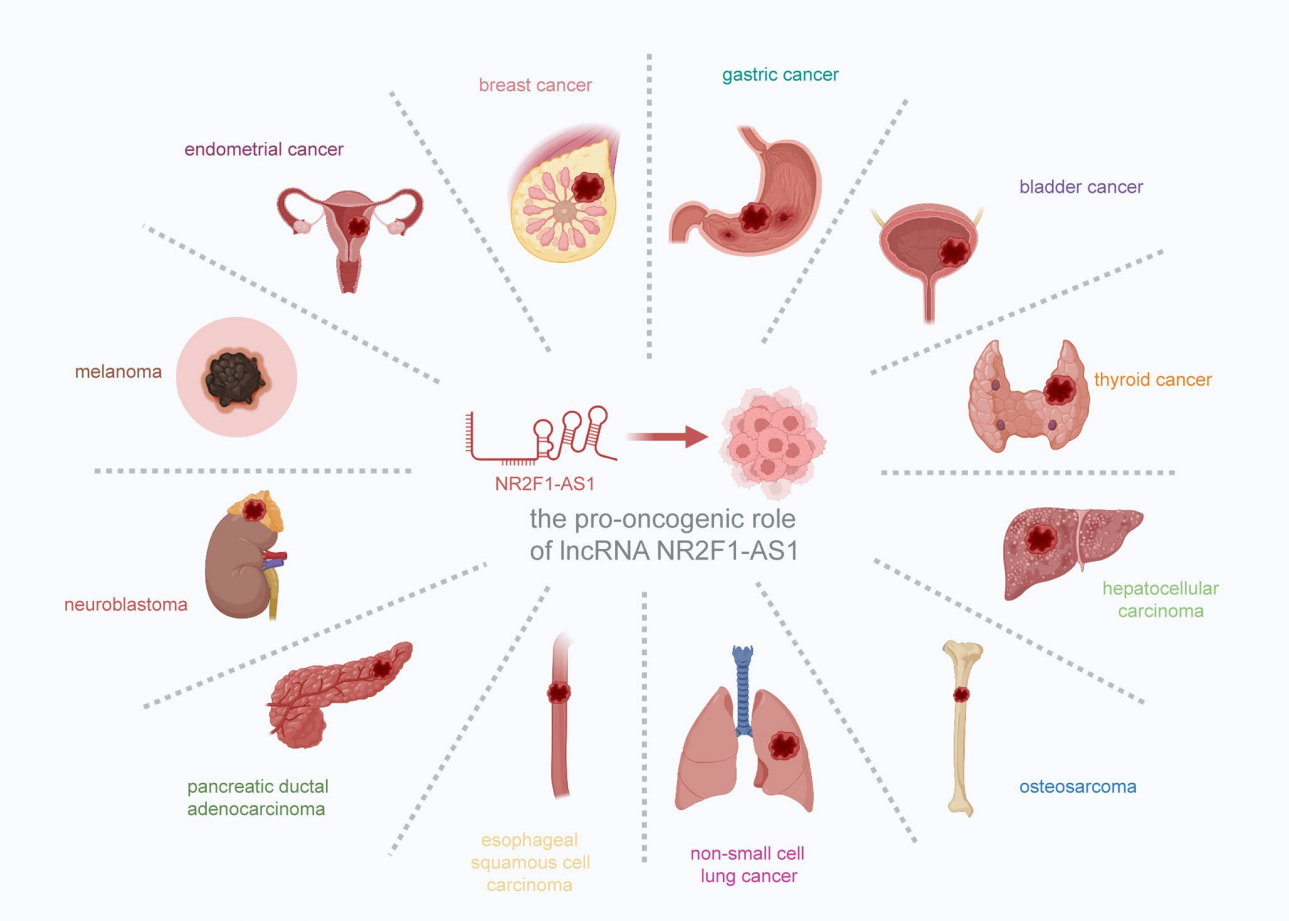
The involvement of NR2F1-AS1 in various human cancer types. The overexpression of NR2F1-AS1 exerts different cancer-promoting roles and has been extensively reported in several human cancers, including breast cancer, gastric cancer, bladder cancer, thyroid cancer, hepatocellular carcinoma, osteosarcoma, non-small cell lung cancer, esophageal squamous cell carcinoma, pancreatic ductal adenocarcinoma, neuroblastoma, melanoma, and endometrial cancer

Recent advances in transcriptomic profiling have revealed that NR2F1-AS1 is aberrantly expressed in a wide array of malignancies, predominantly functioning as an oncogenic lncRNA. Elevated levels of NR2F1-AS1 have been documented in cancers such as breast cancer [25–27], gastric cancer [28–31], bladder cancer [32–34], thyroid cancer [35–37], hepatocellular carcinoma [38–41], non-small cell lung cancer [44], osteosarcoma [42, 43], esophageal squamous cell carcinoma [45, 46], pancreatic ductal adenocarcinoma [53], neuroblastoma [47], melanoma [48], and endometrial cancer [49] (Fig. 1). High expression is frequently associated with unfavorable clinicopathological parameters, including advanced TNM stage, lymph node metastasis, tumor recurrence, and reduced overall and disease-free survival (Table 1).

**Table 1:** NR2F1-AS1 expression and clinical features in human cancers

Disease type	Expression		Clinical characteristics	Refs
breast cancer	upregulated	TNM stage, tumor relapse, lymph node metastasis, total survival time, overall survival, and distant metastasis-free survival		[25–27]
gastric cancer	upregulated	histopathological type, TNM stage, G3 histological grade, perineural invasion, distant metastasis, overall survival, and disease-free survival		[28–31]
bladder cancer	upregulated	poor prognosis		[32–34]
thyroid cancer	upregulated	/		[35–37]
liver cancer	upregulated	overall survival, and pathological stage		[38–41]
osteosarcoma	upregulated	Enneking stage, and distant metastasis		[42, 43]
lung cancer	upregulated	tumor size, TNM stage, lymph node metastasis, and overall survival		[44, 45]
esophageal cancer	upregulated	/		[46, 47]
pancreatic cancer	upregulated	overall survival, and disease-free survival		[54]
neuroblastoma	upregulated	overall survival		[48]
melanoma	upregulated	poor prognosis, and tumor stage		[49]
endometrial cancer	upregulated	/		[50]
colorectal cancer	downregulated	poor survival		[51]
cervical cancer	downregulated	tumor stage		[52]
thyroid cancer	downregulated	overall survival		[53]

Interestingly, while NR2F1-AS1 generally functions as an oncogene, a tumor-suppressive role has been observed in certain cancers, including colorectal cancer, cervical squamous cell carcinoma, and thymic epithelial tumors [50–52]. These findings suggest a context-dependent regulatory function of NR2F1-AS1 that may vary across cancer types.

Functionally, NR2F1-AS1 contributes to tumor progression by modulating key cancer hallmarks such as uncontrolled proliferation, invasion, migration, glycolytic reprogramming, apoptosis resistance, stemness maintenance, and chemoresistance (Table 2). In the following sections, we summarize the expression patterns and clinicopathological relevance of NR2F1-AS1 across various malignancies, along with its functional implications.

**Table 2:** The functions and regulatory mechanisms of NR2F1-AS1 in human cancers

Disease type	Cell lines	Expression	Functions		Related mechanisms		Refs
breast cancer	T47D, BT-474, MCF-7, MDA-MB-468, MDA-MB-231, BT-549, HCC1937, lung-dormant MCF10CA1h, and CA1h-P2	overexpressed	cell proliferation, migration, and invasion		miR-338-3p, miR-205, IGF-1, IGF-1R, ERK, NR2F1, and ΔNp63	[25–27]	
gastric cancer	AGS, SGC7901, HGC-27, KKP, KE-39, MKN-45, MKN-74, SNU-5, and BGC823	overexpressed	cell proliferation, apoptosis, migration, and invasion		TGF-β, miR-29a-3p, VAMP7, miR-190a, PHLDB2, AKT3, SPI1, ST8SIA1, miR-493-5p, and MAP3K2	[28–31]	
bladder cancer	/	overexpressed	cell glycolysis	/		[32]	
thyroid cancer	B-CPAP, FTC‐133, CGTH-W3, K1, and TPC1	overexpressed in thyroid cancer, downregulated in thymic epithelial tumors	cell proliferation, apoptosis, migration, and invasion		miR-338-3p, CCND1, miR-423-5p, and SOX12	[36, 37]	
liver cancer	SNU-398, BEL-7402, HepG2, Hep3B, Huh-7, MHCC97-H, and SK-Hep-1	overexpressed	cell proliferation, invasion, apoptosis, glycolysis, and oxaliplatin resistance		miR-363, miR-140, HK2, miR-642a, DEK, miR-363, and ABCC1	[38–41]	
osteosarcoma	HOS, SAOS-2, MG63, and U2OS	overexpressed	cell proliferation, apoptosis, migration, and invasion		miR-485-5p, miR-218-5p, BIRC5, miR-483-3p, and FOXA1	[42, 43]	
lung cancer	H460, H522, A549, and H1299	overexpressed	cell proliferation, invasion, apoptosis, and glycolysis		miR-493-5p, ITGB1, miR-363-3p, and SOX4	[44, 45]	
esophageal cancer	CA109, TE-1, ECA7906, KYSE-30, KYSE-70, KYSE150, and KYSE450	overexpressed	cell proliferation, migration, invasion, and stemness		NR2F1, and Hedgehog signaling pathway	[46, 47]	
pancreatic cancer	PANC-1, CFPAC-1, Capan-2, SW1990, and BXPC-3	overexpressed	cell proliferation, migration, and invasion		miRNA-146a-5p, and miRNA-877-5p	[54]	
neuroblastoma	SK-N-AS, SK-N-SH, NB-1643, and NB-1691	overexpressed	cell proliferation, apoptosis, migration, and invasion		miR-493-5p, and TRIM2	[48]	
melanoma	SK-MEL-2, K-MEL-28, and A375	overexpressed	cell proliferation, migration, and invasion		STAT3, miR-493-5p, and GOLM1	[49]	
endometrial cancer	HHUA, KLE, Ishikawa, and ECC-1	overexpressed	cell proliferation, apoptosis, migration, and invasion		miR-363, SOX4, PI3K, AKT, and GSK-3β	[50]	
colorectal cancer	RKO	downregulated	cell proliferation		miR-371a-3p, and TOB1	[51]	
cervical cancer	C-33 A	downregulated	cell migration, and invasion		miR-17, and SIK1	[52]	

Breast cancer remains the leading cause of cancer-related morbidity and mortality among women, characterized by high recurrence and metastatic rates [54–58]. Multiple studies have revealed that NR2F1-AS1 is significantly overexpressed in breast cancer tissues and a variety of cell lines, including T47D, BT-474, MCF-7, MDA-MB-468, MDA-MB-231, BT-549, and HCC1937, as well as lung-dormant MCF10CA1h and CA1h-P2 cells [25–27]. Elevated NR2F1-AS1 expression has been linked to advanced TNM stage, tumor relapse, lymph node metastasis, and poorer survival outcomes, such as reduced overall survival and distant metastasis-free survival. Functional investigations have shown that NR2F1-AS1 promotes proliferation, migration, and invasion of MCF-7 cells, facilitates tumor angiogenesis, and enhances tumor growth in mouse and zebrafish models. Interestingly, it also contributes to tumor dissemination while suppressing proliferation in lung tissues, indicating a context-dependent role.

Gastric cancer is one of the most prevalent malignancies of the digestive tract, known for its high aggressiveness and mortality [59–61]. Therefore, elucidating the molecular mechanisms underlying gastric carcinogenesis and identifying sensitive biomarkers remain of great importance [62–65]. Previous studies have demonstrated that NR2F1-AS1 is overexpressed in gastric cancer tissues and multiple cell lines, including AGS, SGC7901, HGC-27, KKP, KE-39, MKN-45, MKN-74, SNU-5, and BGC823 [28–31]. Higher expression levels are associated with poor prognosis and adverse pathological features, including histological subtype, TNM stage, G3 grade, perineural invasion, and reduced overall and disease-free survival. Furthermore, NR2F1-AS1 is considered an EMT-induced gene, capable of enhancing proliferation, inhibiting apoptosis, and promoting migration and invasion in several gastric cancer cell lines, including SGC7901, BGC823, MKN-74, and AGS.

Bladder cancer is a commonly diagnosed malignant tumor of the urinary tract, originating from the bladder mucosa [66–68]. Consequently, identifying novel therapeutic targets is critical for improving survival outcomes in affected patients [69–72]. Numerous studies have confirmed that NR2F1-AS1 is upregulated in bladder cancer tissues and is significantly associated with poor prognosis [32–34]. Functionally, NR2F1-AS1 has been characterized as a glycolysis-related lncRNA, promoting tumor progression through metabolic reprogramming.

Thyroid cancer represents the most frequently diagnosed malignant tumor of the endocrine system, with its incidence steadily increasing in recent years [73–75]. Novel molecular biomarkers have shown potential to enhance diagnosis, prognosis assessment, and treatment strategies for thyroid malignancies [76–79]. NR2F1-AS1 is highly expressed in thyroid cancer tissues and in cell lines such as B-CPAP, FTC‐133, CGTH-W3, K1, and TPC1. Functional studies have shown that it promotes proliferation and migration while suppressing apoptosis in TPC1, K1, FTC-133, and B-CPAP cells [35–37].

**Fig. 2:**
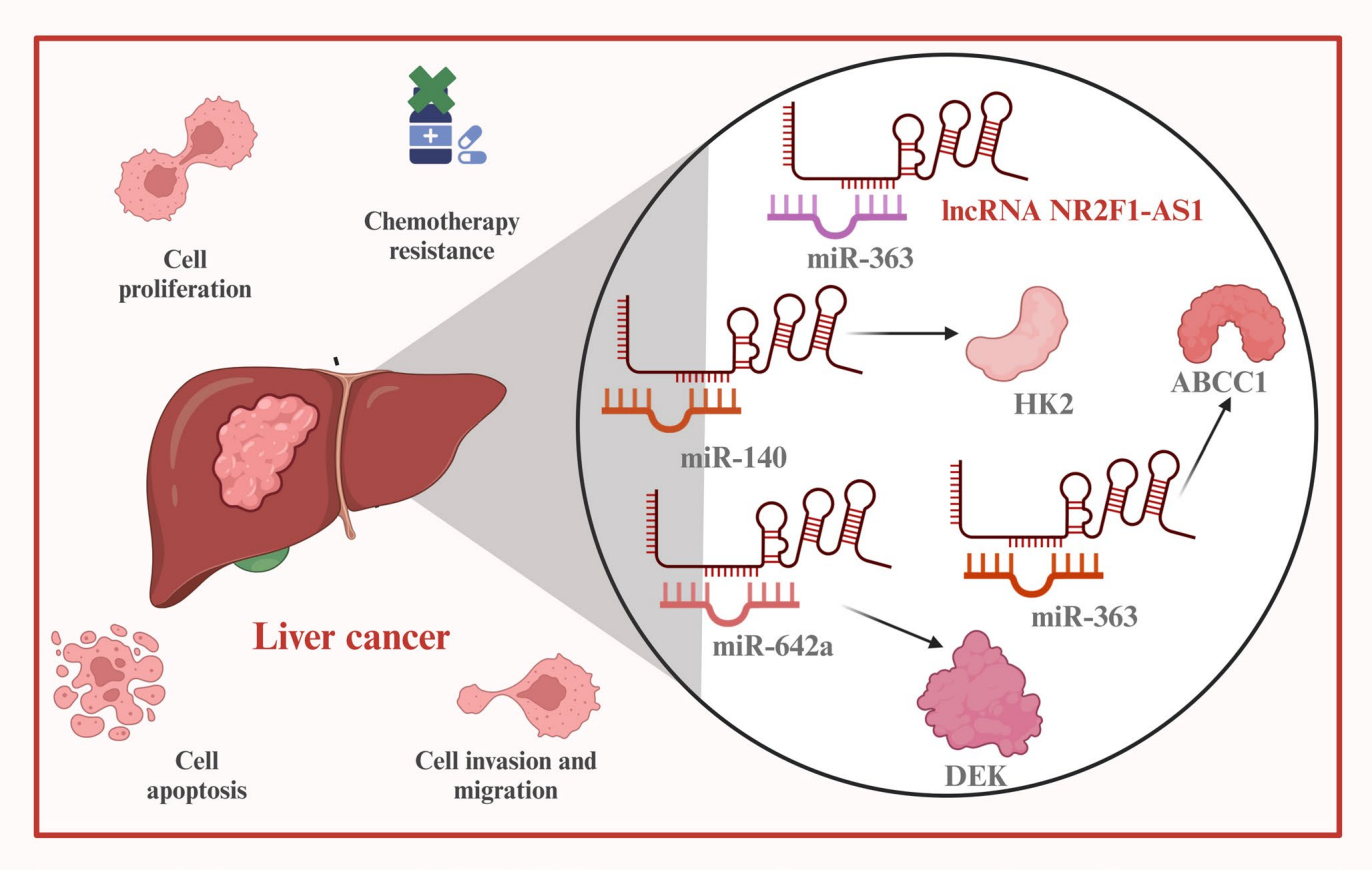
The Roles of NR2F1-AS1 in the progression of hepatocellular carcinoma. In hepatocellular carcinoma, NR2F1-AS1 participates in the regulation of cell proliferation, invasion, apoptosis, and oxaliplatin resistance by combining with miR-363, or via miR-140/HK2, miR-642a/DEK, miR-363/ABCC1 axes

Hepatocellular carcinoma is the most common subtype of primary liver cancer and accounts for approximately 90% of all liver cancer cases, with a consistently high mortality rate [80–83]. Many patients are diagnosed at intermediate or advanced stages, where effective treatment options are limited [84, 85]. Thus, the development of reliable biomarkers for early detection is a top priority [86, 87]. In hepatocellular carcinoma, NR2F1-AS1 is upregulated in tumor tissues, patient serum, and cell lines, including SNU-398, BEL-7402, HepG2, Hep3B, Huh-7, MHCC97-H, and SK-Hep-1. Its expression is positively associated with pathological staging and poor overall survival [38–40]. Functionally, NR2F1-AS1 contributes to proliferation, invasion, apoptosis resistance, glycolysis, and oxaliplatin resistance in cell lines and promotes tumor growth in xenograft models (Fig. 2).

Osteosarcoma is a highly aggressive bone tumor predominantly affecting children and adolescents [88–91]. However, the underlying mechanisms of its progression remain largely undefined [92–95]. Studies have revealed that NR2F1-AS1 is overexpressed in osteosarcoma tissues and cell lines including HOS, SAOS-2, MG63, and U2OS. Its expression is significantly correlated with advanced Enneking stages and distant metastasis. Mechanistically, NR2F1-AS1 promotes cell proliferation, migration, invasion, and inhibits apoptosis in vitro, and accelerates tumor growth in vivo [42, 43].

Non-small cell lung cancer (NSCLC) has the highest incidence and mortality rates globally, accounting for a large proportion of cancer-related deaths [96–98]. Due to its dismal 5-year survival rate (below 15%), identifying early diagnostic and therapeutic biomarkers remains urgent [99–102]. NR2F1-AS1 is highly expressed in NSCLC tissues and cell lines such as H460, H522, A549, and H1299 [44]. Its expression correlates with tumor size, TNM staging, lymph node metastasis, and overall survival. Functionally, NR2F1-AS1 facilitates cancer development by regulating apoptosis, proliferation, glycolysis, migration, and invasion in vitro and promotes tumor growth in xenograft models.

Esophageal cancer is among the most lethal malignancies worldwide, known for its aggressiveness and poor prognosis [103–106]. Recent studies have highlighted the critical roles of lncRNAs in the progression of esophageal squamous cell carcinoma (ESCC) [107–109]. NR2F1-AS1 is overexpressed in ESCC tissues and multiple cell lines, including CA109, TE-1, ECA7906, KYSE-30, KYSE-70, KYSE-150, and KYSE-450. It acts as a tumor promoter by enhancing proliferation, metastasis, and stemness features of KYSE150, KYSE450, ECA109, and TE-1 cells [45, 46].

Furthermore, NR2F1-AS1 is also important in other cancers. In pancreatic ductal adenocarcinoma, NR2F1-AS1 is upregulated in PANC-1, CFPAC-1, Capan-2, SW1990, and BXPC-3 cells and correlates with poor overall and disease-free survival [53]. In this case, NR2F1-AS1 contributed to cancer progression by promoting the proliferation, migration, and invasion of PANC-1 and CFPAC-1 cells, and tumorigenesis in a mouse model. In neuroblastoma, high NR2F1-AS1 levels are observed in tissues and SK-N-AS, SK-N-SH, NB-1643, and NB-1691 cells. It correlates with poor prognosis and exerts oncogenic effects by enhancing proliferation, invasion, and suppressing apoptosis in SK-N-SH and SK-SY5Y cells [47]. Similarly, NR2F1-AS1 is overexpressed in melanoma tissues and cell lines (SK-MEL-2, K-MEL-28, A375), promoting proliferation, migration, and invasion, and correlating with poor clinical outcomes [48]. In endometrial cancer, it is upregulated in HHUA, KLE, Ishikawa, and ECC-1 cells and enhances tumor progression by promoting proliferation and inhibiting apoptosis [49].

On the other hand, NR2F1-AS1 is downregulated in colorectal cancer RKO cells and is associated with poor patient survival. NR2F1-AS1 act as a tumor suppressor by inhibiting the proliferation of RKO cells. In cervical squamous cell carcinoma, NR2F1-AS1 is also reduced in tumor tissues and C-33 A cells, correlating with advanced tumor stages. It suppresses invasion and migration in C-33 A cells [51]. Similarly, in thymic epithelial tumors, downregulation of NR2F1-AS1 is associated with worse overall survival [52].

### Mechanisms underlying NR2F1-AS1 functions in cancers

NR2F1-AS1 is implicated in a variety of cellular processes central to cancer development and progression. Through its interaction with miRNAs and key transcriptional regulators, it modulates cancer hallmarks including proliferation, apoptosis, glycolysis, migration, invasion, stemness, and chemoresistance. Among these, its roles in regulating proliferation and metastatic behavior have been the most intensively investigated.

### Cell proliferation

Uncontrolled cell proliferation, largely driven by dysregulation of the cell cycle, is a hallmark of tumorigenesis [110–113]. NR2F1-AS1 has been widely reported to promote proliferation in various malignancies through ceRNA mechanisms, epigenetic modulation, and transcriptional activation. In breast cancer, NR2F1-AS1 induces dormancy in disseminated tumor cells by suppressing ΔNp63 via NR2F1/miR-205 axis [26, 27]. In gastric cancer, NR2F1-AS1 enhances cell growth through various axes, including miR-29a/VAMP7 [28], miR-190a/PHLDB2/AKT3 [29], SPI1/ST8SIA1 [30], and miR-493-5p/MAP3K2 [31]. In thyroid cancer, NR2F1-AS1 promotes proliferation and inhibits apoptosis via the miR-338-3p/CCND1 and miR-423-5p/SOX12 pathways [36, 37]. In hepatocellular carcinoma, it acts through miR-363 regulation to stimulate cell division in BEL-7402 and Huh-7 cells [38]. In osteosarcoma, NR2F1-AS1 accelerates proliferation and apoptosis resistance through both the miR-485-5p/miR-218-5p/BIRC5 and miR-483-3p/FOXA1 axes [42, 43]. Similarly, in non-small cell lung cancer, it enhances cell cycle progression via miR-363-3p/SOX4 signaling [44]. In esophageal squamous cell carcinoma, NR2F1-AS1 enhances proliferation of ECA109, TE-1, KYSE150, and KYSE450 cells by activating the Hedgehog signaling pathway [46]. In pancreatic ductal adenocarcinoma, NR2F1-AS1 promotes proliferation via inhibition of miR-146a-5p and miR-877-5p [53]. Neuroblastoma cells (SK-N-SH and SK-SY5Y) exhibit increased proliferation and reduced apoptosis upon NR2F1-AS1–mediated activation of TRIM2 via miR-493-5p [47]. In melanoma, NR2F1-AS1 is transcriptionally activated by STAT3 and drives proliferation by repressing miR-493-5p and enhancing GOLM1 [48]. In endometrial cancer, it promotes proliferation and anti-apoptotic effects by sponging miR-363, upregulating SOX4, and activating the PI3K/AKT/GSK-3β pathway [49].

Conversely, NR2F1-AS1 acts as a tumor suppressor in colorectal cancer by sponging miR-371a-3p, leading to increased TOB1 expression and subsequent inhibition of proliferation in RKO cells [50] Similarly, in cervical squamous cell carcinoma, it suppresses proliferation by interacting with miR-17 to enhance SIK1 expression [51].

Collectively, these findings suggest that NR2F1-AS1 exerts context-specific effects on cancer cell proliferation, predominantly by altering miRNA-mediated gene expression and modulating key pathways that drive cell cycle progression.

### Cell invasion and migration

**Fig. 3:**
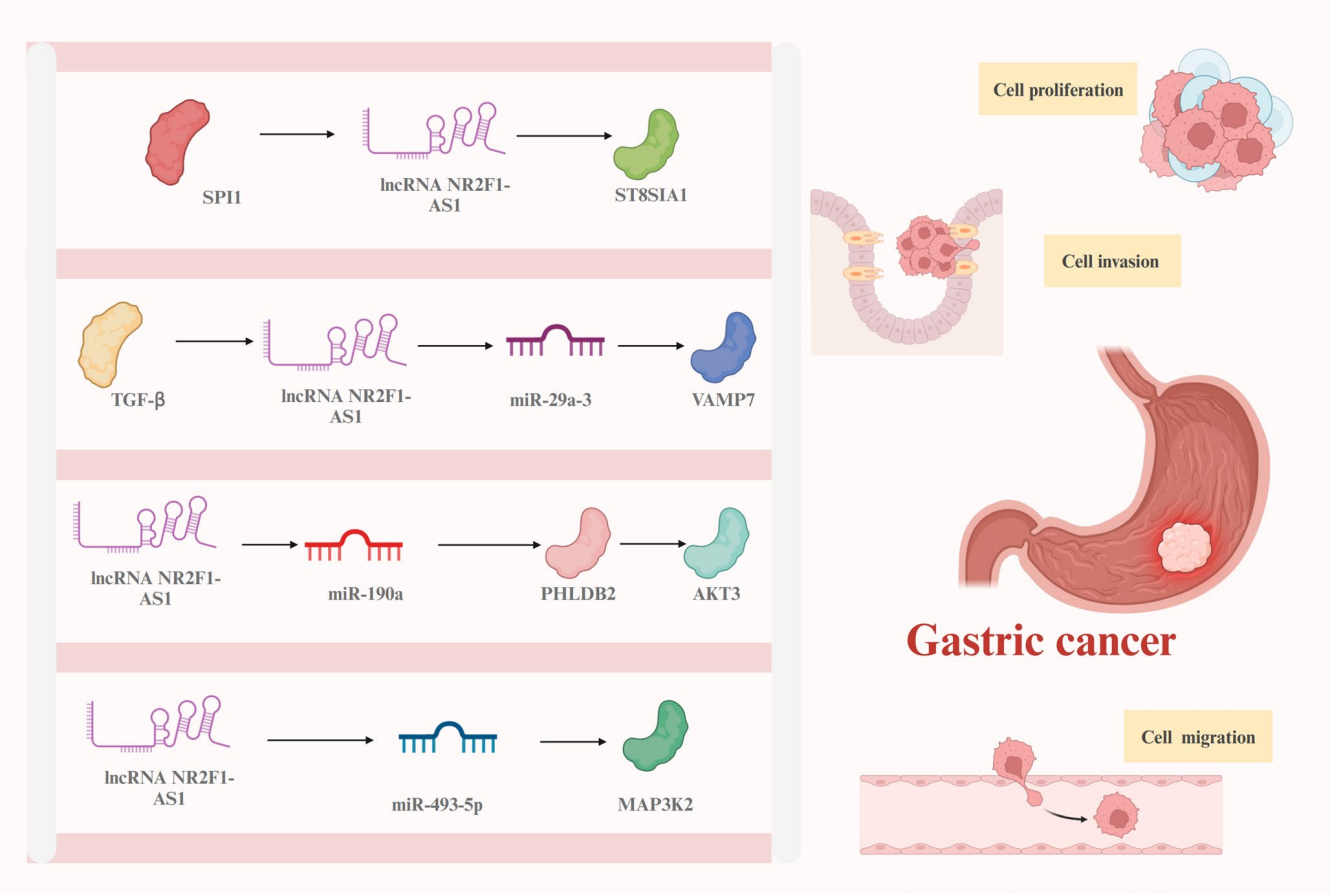
The regulatory mechanisms of NR2F1-AS1 in gastric cancer. In gastric cancer, NR2F1-AS1 plays pro-oncogenic roles via miR-29a sponging to increase the expression of VAMP7; miR-190a sponging to upregulate PHLDB2 and subsequently activate the expression of AKT3; by binding to SPI1 to increase the expression of ST8SIA1; by interacting with miR-493-5p to increase the expression of MAP3K2

While proliferation governs cellular expansion in situ, migration and invasion reflect a tumor’s ability to disseminate, breach tissue barriers, and form metastases, processes that are mechanistically distinct and clinically more lethal [114–117]. NR2F1-AS1 contributes to cancer invasiveness by regulating epithelial–mesenchymal transition (EMT), extracellular matrix (ECM) remodeling, and cell motility through a network of miRNA interactions and signaling modulation. In breast cancer, NR2F1-AS1 facilitates the migration and invasion of MCF-7 cells via activation of the IGF-1/IGF-1R/ERK signaling cascade through miR-338-3p [25]. It further promotes metastatic potential by increasing NR2F1 translation and downregulating ΔNp63, thereby supporting dissemination in MCF-7 and T47D cells [26, 27]. In gastric cancer, NR2F1-AS1 acts as an EMT-inducing lncRNA that enhances cell motility via the miR-29a/VAMP7 axis, activation of AKT3 through miR-190a/PHLDB2, SPI1-mediated transcriptional upregulation of ST8SIA1, and miR-493-5p/MAP3K2 signaling (Fig. 3) [28–31]. These mechanisms not only facilitate migration but also promote cytoskeletal reorganization and basement membrane degradation. In thyroid cancer, its migratory function is executed through similar ceRNA interactions, targeting miR-338-3p/CCND1 and miR-423-5p/SOX12 [36, 37]. In hepatocellular carcinoma, NR2F1-AS1 promotes invasive behaviors by modulating glycolysis- and metastasis-related targets such as HK2, DEK, and ABCC1 through miR-140, miR-642a, and miR-363 [38–41]. In osteosarcoma, NR2F1-AS1 enhances cell motility through miR-483-3p/FOXA1 and miR-485-5p/miR-218-5p/BIRC5 pathways, contributing to ECM degradation and metastasis [42, 43]. In non-small cell lung cancer, NR2F1-AS1 enhances migration and invasion through upregulation of SOX4 by targeting miR-363-3p [44]. In esophageal squamous cell carcinoma, its effects on invasion are mediated through Hedgehog pathway activation [46], while in pancreatic ductal adenocarcinoma, migration is enhanced via suppression of miR-146a-5p and miR-877-5p [53]. In neuroblastoma, NR2F1-AS1 enhances motility in SK-N-SH and SK-SY5Y cells through the miR-493-5p/TRIM2 axis [47]. In melanoma cells (SK-MEL-2 and A375), STAT3-mediated upregulation of NR2F1-AS1 leads to the activation of the miR-493-5p/GOLM1 axis, thereby promoting invasion and migration [47]. In endometrial cancer, NR2F1-AS1 promotes migratory potential by targeting miR-363/SOX4 and activating the PI3K/AKT/GSK-3β pathway [49].

In summary, while some upstream molecules overlap with those regulating proliferation, the downstream phenotypic outcomes in migration and invasion are mediated through distinct signaling pathways, especially those involving EMT markers, cell–ECM interaction, and cytoskeletal remodeling. These findings establish NR2F1-AS1 as a key regulator of cancer metastasis across diverse tumor types.

### Potential of NR2F1-AS1 for clinical applications

**Fig. 4:**
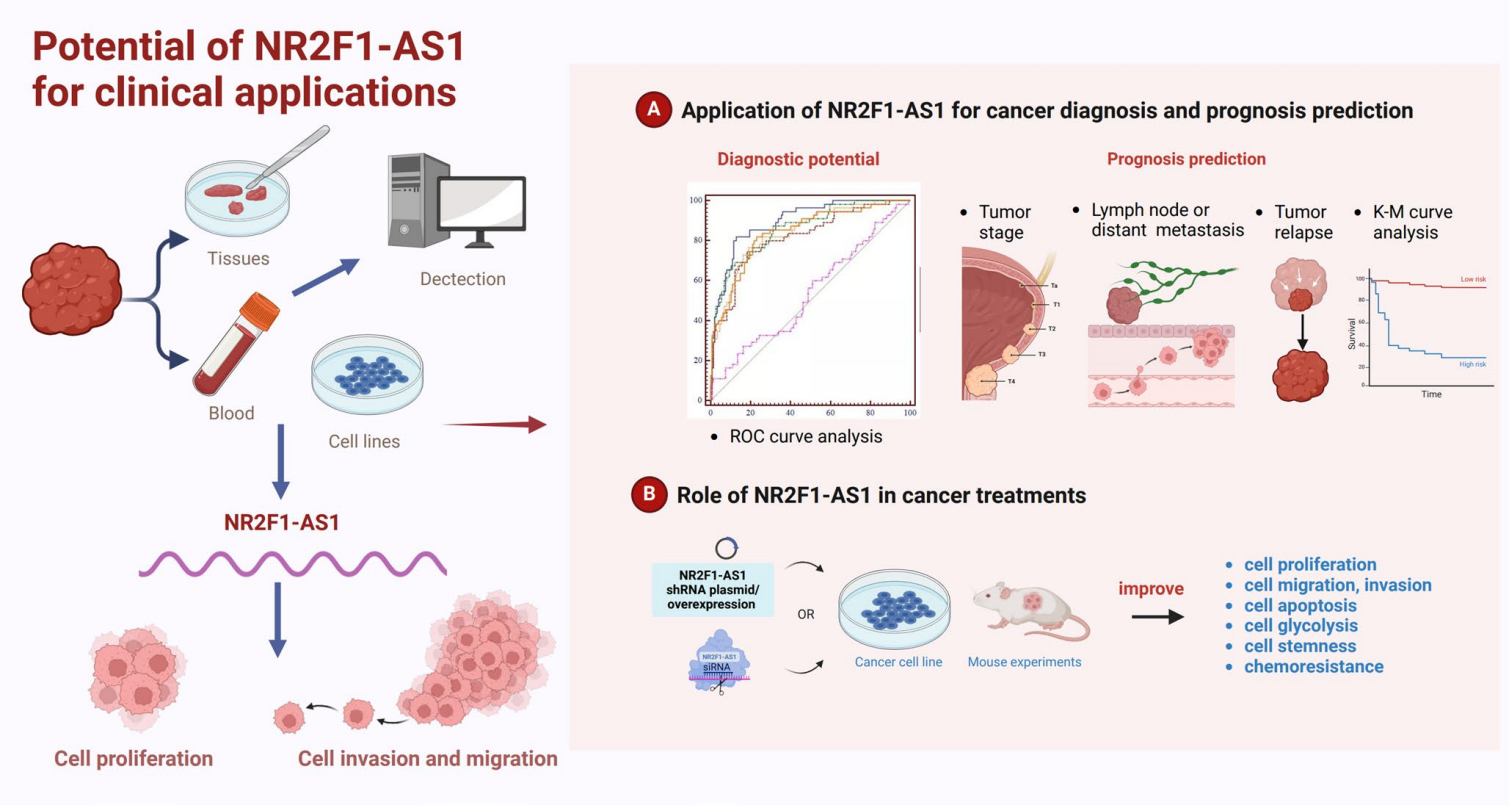
The clinical relevance of NR2F1-AS1 in human cancers. NR2F1-AS1 is aberrantly expressed in various tumor-derived samples, including tissues, blood samples, and established cell lines. Its dysregulation contributes to enhanced cancer cell proliferation, migration, and invasion. ROC curve analysis demonstrates the diagnostic accuracy of NR2F1-AS1, while its expression is associated with clinical parameters such as tumor stage, lymph node or distant metastasis, and tumor recurrence. Kaplan–Meier (K-M) survival analysis supports its use in stratifying patients by risk. Functional assays using cancer cell lines and in vivo mouse models show that modulation of NR2F1-AS1 expression impacts key malignant phenotypes, underscoring its value as a potential therapeutic target

Extensive functional analyses have demonstrated that NR2F1-AS1 plays a pivotal role in modulating a wide range of tumor-related biological processes. As such, NR2F1-AS1 holds significant potential for diverse clinical applications, including cancer diagnosis, prognosis prediction, and therapeutic intervention (Fig. 4). These aspects are elaborated upon in the following sections.

### Application of NR2F1-AS1 for cancer diagnosis and prognosis prediction

Recent studies have revealed marked differences in NR2F1-AS1 expression between malignant and normal tissues or cell lines, thereby laying a solid foundation for its use in cancer diagnosis. For instance, in melanoma, receiver operating characteristic (ROC) curve analysis validated the diagnostic potential of NR2F1-AS1, achieving an area under the curve (AUC) value of 0.7365 for distinguishing tumor from normal samples [48]. These findings suggest that quantifying NR2F1-AS1 expression in tissue specimens may offer a valuable tool for cancer detection.

While the majority of current research has concentrated on tissue and cellular expression, recent evidence also indicates elevated levels of NR2F1-AS1 in the serum of hepatocellular carcinoma (HCC) patients [38]. Considering the advantages of serum-based assays, such as minimal invasiveness, easy accessibility, real-time monitoring, and high reproducibility [118–121], serum NR2F1-AS1 detection presents a promising avenue for early cancer diagnosis.

In addition to diagnostic value, NR2F1-AS1 has demonstrated strong associations with clinicopathological features and poor patient prognosis across multiple cancer types, suggesting its utility as a prognostic biomarker. For example, in bladder cancer, NR2F1-AS1 has been identified as a glycolysis-related lncRNA capable of stratifying patients into high- and low-risk groups, with robust predictive performance as validated by ROC curve analysis [32–34]. Similarly, in thymic epithelial tumors, NR2F1-AS1 demonstrated favorable prognostic value with an AUC exceeding 0.65. Collectively, these findings underscore the prognostic potential of NR2F1-AS1, supporting its use in clinical risk stratification and disease outcome prediction.

### Role of NR2F1-AS1 in cancer treatments

Given its functional involvement in key oncogenic processes, including cell proliferation, migration, invasion, apoptosis, glycolysis, stemness, and chemoresistance, NR2F1-AS1 has emerged as a promising therapeutic target across multiple cancer types. Accumulating mechanistic evidence provides a strong theoretical foundation for its inclusion in oncological treatment strategies. For instance, NR2F1-AS1 has been implicated in the enhancement of chemoresistance by promoting metastasis and facilitating long-term dormancy of disseminated tumor cells. In estrogen receptor (ER)-positive breast cancer, elevated NR2F1-AS1 expression has been associated with a higher risk of recurrence following endocrine therapy [27]. Functional studies have shown that downregulation of NR2F1-AS1 can attenuate the malignant phenotype of dormant breast cancer cells and sensitize them to chemotherapeutic agents, thereby improving therapeutic efficacy [26, 27]. Moreover, targeting metabolic vulnerabilities such as glycolysis has been increasingly recognized as an effective strategy in managing NSCLC. NR2F1-AS1 has been shown to enhance glycolytic flux in A549 and H522 cells by sponging miR-363-3p and subsequently upregulating SOX4 expression [44]. Functional knockdown of NR2F1-AS1 using short hairpin RNA (shRNA) in these cell lines has demonstrated a marked reduction in the expression of glycolysis-associated genes, as well as overall metabolic activity. Notably, future studies employing gene-editing techniques such as CRISPR-Cas9 to generate NR2F1-AS1 knockout (KO) models could offer more definitive insights and potentially reveal stronger glycolytic suppression, thereby enhancing therapeutic efficacy.

In summary, current evidence strongly suggests that modulating NR2F1-AS1 expression, through silencing or potential gene knockout, could effectively disrupt crucial tumor-promoting pathways. These approaches may represent a novel and viable strategy to enhance cancer treatment responses and overcome drug resistance in selected cancers.

## Conclusions and perspectives

A growing body of research has reported the dysregulated expression of NR2F1-AS1 across various human malignancies, including breast cancer, gastric cancer, bladder cancer, thyroid cancer, esophageal squamous cell carcinoma, endometrial cancer, osteosarcoma, hepatocellular carcinoma, non-small cell lung cancer, pancreatic ductal adenocarcinoma, neuroblastoma, melanoma, colorectal cancer, cervical squamous cell carcinoma, and thymic epithelial tumors. To date, NR2F1-AS1 expression has been well-characterized in tumor tissues and cancer cell lines. However, its expression profiles in non-invasive clinical specimens, such as peripheral blood and other body fluids, remain largely unexplored. Further investigations are therefore warranted to assess the detectability, expression stability, and diagnostic value of NR2F1-AS1 in non-invasive sample types, which may pave the way for its clinical application as an early diagnostic biomarker. The strong correlation between NR2F1-AS1 expression and various clinicopathological features across multiple cancer types also highlights its potential as a reliable prognostic indicator.

In addition to its diagnostic and prognostic value, NR2F1-AS1 participates in multiple tumor-related biological processes, including cell proliferation, resistance to apoptosis, migration, invasion, and chemoresistance. Mechanistically, its function is largely mediated through interactions with miRNAs and transcriptional networks. However, accumulating evidence also suggests that the role of NR2F1-AS1 is highly context-dependent, functioning as an oncogenic driver in many cancers (e.g., breast, lung, liver, gastric), while potentially serving as a tumor suppressor in others, such as colorectal cancer, cervical squamous cell carcinoma, and thymic epithelial tumors. This dual role appears to be determined by cancer type–specific regulatory axes, downstream targets, and the tumor microenvironment. Therefore, future studies should focus on systematically characterizing the context-specific functional landscape of NR2F1-AS1 in different cancers. Such efforts will not only clarify its biological roles but also inform the rational design of targeted interventions. Additionally, the development of NR2F1-AS1-based therapeutic strategies, such as antisense oligonucleotides, siRNAs, or gene-editing tools, will require a deep understanding of its safety, specificity, and efficacy in clinical applications.

Looking ahead, NR2F1-AS1 represents a promising candidate for cancer diagnosis, prognosis, and therapy. However, realizing its full clinical potential will depend on a nuanced appreciation of its dual functional roles and cancer type–specific regulatory mechanisms.

## Data Availability

No datasets were generated or analysed during the current study.
